# The new trauma score (NTS): a modification of the revised trauma score for better trauma mortality prediction

**DOI:** 10.1186/s12893-017-0272-4

**Published:** 2017-07-03

**Authors:** Jin Hee Jeong, Yong Joo Park, Dong Hoon Kim, Tae Yun Kim, Changwoo Kang, Soo Hoon Lee, Sang Bong Lee, Seong Chun Kim, Daesung Lim

**Affiliations:** 1 Department of Emergency Medicine, Gyeongsang National University School of Medicine, 15, Jinju-daero 816beon-gil, Jinju-si, Gyeongsangnam-do 52727 Republic of Korea; 2 Department of Emergency Medicine, Gyeongsang National University School of Medicine and Gyeongsang National University Changwon Hospital, Changwon, Republic of Korea; 3 Institute of Health Sciences, Gyeongsang National University, 15, Jinju-daero 816beon-gil, Jinju-si, Gyeongsangnam-do Republic of Korea

**Keywords:** Trauma severity indices, Injury severity score, Emergency department

## Abstract

**Background:**

Since its introduction, the Revised Trauma Score (RTS) has been widely used to determine the prognosis of trauma patients. Recent studies have revealed a need to change the parameters of the RTS. We have designed a new trauma score (NTS) based on revised parameters, including the adoption of the actual Glasgow Coma Scale (GCS) score instead of a GCS code, the revision of the systolic blood pressure interval used for the code value and the incorporation of peripheral oxygen saturation (SpO_2_) instead of respiratory rate. The purpose of this study was to evaluate the predictive performance of the NTS for in-hospital mortality compared with the RTS and other trauma scores.

**Methods:**

This was a prospective observational study using data from the trauma registry of a tertiary hospital. The subjects were selected from patients who arrived at the ED between July 1, 2014, and June 30, 2016, and, for external validation purposes, those who arrived at the ED between July 1, 2011, and June 30, 2013. Demographic data and physiological data were analyzed. NTS models were calculated using logistic regression for GCS score, SBP code values, and SpO_2_. The mortality predictive performance of NTS was compared with that of other trauma scores.

**Results:**

A total of 3263 patients for derivation and 3106 patients for validation were included in the analysis. The NTS showed better discrimination than the RTS (AUC = 0.935 vs. 0.917, respectively, AUC difference = 0.018, *p* = 0.001; 95% CI, 0.0071–0.0293) and similar discrimination to that of mechanism, Glasgow Coma scale, age, and arterial pressure (MGAP) and the Glasgow Coma Scale, age, and systolic arterial pressure (GAP). In the validation cohort, the global properties of the NTS for mortality prediction were significantly better than those of the RTS (AUC = 0.919 vs. 0.906, respectively; AUC difference = 0.013, *p* = 0.013; 95% CI, 0.0009–0.0249) and similar to those of the MGAP and GAP.

**Conclusions:**

The NTS predicts in-hospital mortality substantially better than the RTS.

## Background

In the 30 years since Champion et al. introduced the Revised Trauma Score (RTS), it has been widely used to assess prognosis in trauma patients. The RTS is a convenient tool for trauma triage and initial severity estimation that does not require sophisticated medical tests or devices and is especially useful in prehospital and emergency department (ED) settings. This physiological scoring system consists of the Glasgow Coma Scale (GCS), systolic blood pressure (SBP) and respiratory rate (RR). The parameters are converted to coded values (0, 1, 2, 3 or 4) assigned by specified ranges. Each value is multiplied by a weighted coefficient before it is added (Table 1) [1]. The RTS is calculated using the following equation:

**Table 1:** Modification of the Revised Trauma Score

Revised Trauma Score				New Trauma Score		
Glasgow Coma Scale	Systolic Blood Pressure	Respiratory Rate	Coded Value	Glasgow Coma Scale	Systolic Blood Pressure	Oxygen saturation
13–15	>89	10–29	4	3–15	110–149	≥94
9–12	76–89	>29	3		≥150	80–93
6–8	50–75	6–9	2		90–109	60–79
4–5	1–49	1–5	1		70–89	40–59
3	0	0	0		<70	<40

RTS = (0.9368 x GCS code value) + (0.7326 x SBP code value) + (0.2908 x RR code value).

The Trauma and Injury Severity Score (TRISS), developed in 1987 by Boyd et al., has been used worldwide to predict trauma survival. The TRISS consists of physiological (RTS) and anatomical scores (Injury Severity Score, ISS) and age, stratified by the injury mechanism (blunt or penetrating trauma) [2]. The TRISS showed substantially improved predictive power of survival for trauma patients over that of the RTS and was validated in subsequent studies [3, 4]. Despite the complicated calculation and its inapplicability for triage, the TRISS remains the most widely used and prominent survival predictor for research in the quality control of trauma management and prevention.

The Mechanism, GCS, and Age and Arterial Pressure (MGAP) score is a recently developed physiological trauma scoring system. Similar to the TRISS, the MGAP utilized mechanism and age, which are important variables that affect the prognosis of trauma patients. The final scores can be easily obtained after the simple addition of several numbers. While the GCS is transformed to a code value ranging from 0 to 4 in the RTS, the MGAP consists of the actual GCS score with no variation due to its highly informative value and relatively unbiased calculation. Additionally, a SBP of 120 mmHg was chosen for the threshold, whereas 90 mmHg is the first cutoff for decreasing the code value in the RTS [5]. The GCS, Age and Systolic Arterial Pressure (GAP) is a scoring system that was simplified by deleting the mechanism from the MGAP [6]. The MGAP and GAP were shown to be superior to the RTS in mortality prediction of trauma patients. However, they remain inferior to the TRISS.

Uncontrolled hemorrhage is a major leading cause of traumatic injury that is responsible for 35% of prehospital deaths and over 40% of deaths within the first 24 h [7]. Traditionally, a SBP of <90 mmHg has been a widely accepted threshold for hypotension. The American College of Surgeons Committee on Trauma and National Expert Panel on Field Triage recommends a SBP of <90 mmHg for specialized trauma centers [8, 9]. Recently, the concept of a SBP of <90 mmHg as an early indicator of hypotension in trauma patients has become controversial. SBPs of 90–109 mmHg in the ED or the operating room result in worse outcomes than higher SBPs [10]. Furthermore, in a large population cohort study using data from the Trauma Audit and Research Network (*n* = 47,927), a SBP of <110 mmHg was identified as a cut off for hypotension, at which a significant increase in mortality occurred [11].

Instead of the recommended formal measurement technique of 1 min, the RR is commonly assessed during a short period of less than 30 s [12]. In the prehospital and ED settings, exact RR measurement by auscultation for 1 min is challenging due to patient conditions, loud noises and psychological or emotional pressures on medical personnel. Short RR counting for a 30-s interval is naturally inaccurate compared to a one-minute interval [13]. Recent research has shown that RR measurements obtained by triage nurses using an electronic device in the ED are inaccurate [14]. The same results were found in another study of medical doctors working at a teaching hospital who had been taught accurate measurement techniques immediately before the study [15]. Pulse oximetry is a popular monitoring method widely used in various settings [16–20]. Peripheral oxygen saturation (SpO_2_) is an objective, efficient and unequivocal parameter for screening patient pulmonary function [21, 22]. Practically, SpO_2_ has become a substitute for the RR in the past decade. Accordingly, we speculated that SpO_2_ could be a better component than the RR for trauma mortality prediction.

The RTS is a widely valued but somewhat outdated scoring system for trauma mortality prediction. Therefore, we modified the RTS and designed a new trauma score based on recent developments in the trauma setting. The main ideas include (i) the adoption of the actual GCS score instead of a GCS code, (ii) the revision of the systolic blood pressure interval used for the code value and (iii) the incorporation of SpO_2_ instead of RR. The details are represented in Table 1. We termed this measure the New Trauma Score (NTS).

The purpose of this study was to evaluate the predictive performance of the NTS for in-hospital mortality compared to the RTS, MGAP and GAP as well as to provide a proper triage tool during the initial phase of trauma management.

## Methods

### Study design and participants

This is a prospectively recorded registry-based observational study using data from the trauma registry of a tertiary hospital located in Jinju, Republic of Korea. Data collection started on July 1, 2011 and was recorded by professional heath information managers in our ED. Demographic data, age, gender and physiological data regarding the initial presentation to the ED, as well as outcome and in-hospital mortality, were automatically transferred to the trauma registry from the electronic medical record. Injury mechanisms were categorized as blunt trauma, penetrating trauma, burn, drowning, hanging, asphyxia, poisoning and heat or cold-related injury. Abbreviated Injury Scales (AIS) were calculated according to clinical presentation, imaging results, intervention findings and operative records. Injury descriptions and scoring procedures were fully supervised by emergency physicians. The trauma registry was originally developed as a part of Emergency Department-based Injury In-depth Surveillance conducted by the Korea Centers for Disease Control and Prevention. Informed consent was not needed because the data were collected without identifiable personal information. Data used for derivation were obtained from patients arriving in the ED between July 1, 2014 and June 30, 2016. The inclusion criteria consisted of (i) patients categorized with blunt or penetrating mechanisms and (ii) age ≥ 15 years. The exclusion criteria consisted of (i) patients who died before ED arrival, (ii) patients discharged or transferred from the ED. We used the data from patients arriving at the same hospital between July 1, 2011 and June 30, 2013 for external validation purposes. This study was approved by the Gyeongsang National University hospital institutional review board (number 2016–09-008).

### Development of NTS

**Fig. 1:**
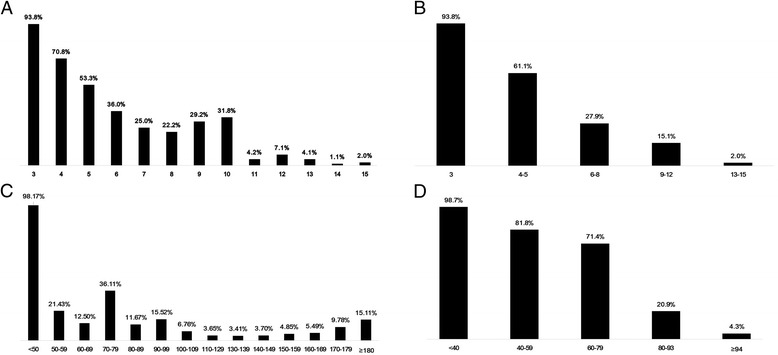
The mortality according to variables for the New Trauma Score. **a** Actual Glasgow Coma Scale score. **b** Coded value of Glasgow Coma Scale. **c** Systolic blood pressure. **d** Oxygen saturation

To compare the value of the GCS codes used in the RTS and the actual GCS score, we plotted frequency charts for in-hospital mortality (Fig. 1a, b). Using the group with a score of 4 as a reference, the odds ratios of GCS codes of 3, 2, 1, and 0 were calculated as categorical variables in the univariate regression. The actual GCS scores were entered into the logistic regression as continuous variables. Both parameters were significant and were used in the next step.

The patient distribution showed a bimodal rather than linear correlation between SBP and death (Fig 1c). We chose 110–149 mmHg as the range for a score of 4, and SBP ≥ 150 mmHg received a score of 3. Blood pressure measurement of trauma victims is very challenging in prehospital or ED triage settings, especially when patients are exsanguinating and progressing to profound shock. It might be impractical for clinical decision making if the interval boundaries are set too low. For that reason, we chose 70 mmHg as the lowest cutoff, and half of the interval (SBP 90 mmHg) was set as another cut-point. The SpO_2_ was divided into five categories, ≥ 94%, 80–93%, 60–79%, 40–59% and <40% (Fig 1d). Because no references are available to determine SpO_2_ interval ranges related to trauma mortality prediction, we must depend on clinical experience.

### Data analysis

We performed multiple imputation using multivariate imputation by chained equation (MICE) to impute missing values [23]. The number of multiple imputations should reach the percentage of the missing proportion [24]. In our data set, the ISS was the most frequently missing variable (8.9% and 7.9% in the derivation and validation cohorts, respectively), followed by SpO_2_ (3.2% and 2.8%), SBP, RR, and GCS (not exceeding 0.1% in both cohorts). Therefore, we conducted ten imputations using a predictive mean-matching method for all variables included in the RTS, MGAP, GAP, and the NTS. Age, gender, the ISS and the outcome variable (in-hospital mortality) were also included in the imputation model.

The χ^2^ test and the Mann-Whitney *U* test (because all the continuous data showed non-normal distribution) were used to describe demographic and physiological characteristics of the derivation and validation groups.

A univariate analysis was performed on the GCS score, SBP, RR, SpO_2_, and the coded GCS value. Multivariate logistic regression was conducted using the GCS score, SBP and RR, SpO_2_ as continuous variables and using the GCS score, SBP and SpO_2_. We categorized the SBP and SpO_2_ and assigned code values of 0, 1, 2, 3 or 4 (Table 1). Multivariate logistic regression was performed using the actual GCS score, the coded SBP (SBP_NTS_) and SpO_2_ (SpO_2NTS_) values, and the coded GCS, SBP_NTS_, and SpO_2NTS_. Predictive survival (Ps) was calculated using the following equation: Ps = 1 / (1 + e^-b^), b = b_0_ + b_1_ x GCS + b_2_ x SBP_NTS_ + b_3_ x SpO_2NTS_. b_0_, b_1_, b_2_ and b_3_ were coefficients derived in the regression analysis. The discriminatory ability of the final models was assessed using the receiver operating characteristics (ROC) curve, and calibration was evaluated using the Hosmer-Lemeshow (H-L) statistic. The areas under the ROC curve (AUC) were compared between the NTS, RTS, MGAP and GAP using the nonparametric approach described by DeLong et al. [25]. We deleted the regression coefficients from the NTS to provide an easy triage tool called the NTS for Triage (T-NTS).

The sensitivity and specificity were obtained from the point of the ROC curve. The specificity of NTS was compared to those of RTS, MGAP, GAP, respectively, at the point at which their sensitivity reached 95% [4, 10]. We divided patients into four groups according to the T-NTS and observed mortality in each group. The final model was validated with separate data using ROC curve analysis to compare our method with the RTS, MGAP and GAP.

All *p* values were two-sided, and a value of *p* < 0.05 was considered statistically significant. Analyses were performed using MedCalc 17 (MedCalc Software BVBA, Ostend, Belgium) and Stata version 13 (StataCorp, LP, College Station, TX).

## Results

### Baseline characteristics

**Fig. 2:**
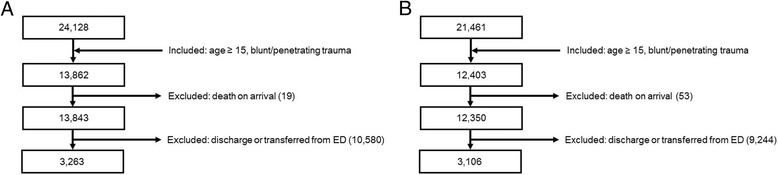
Patient flow according to inclusion and exclusion criteria. **a** Derivation cohort. **b** Validation cohort

A total of 24,128 patients were enrolled in the Gyeongsang National University Hospital Trauma Registry between January 2014 and June 2016. Among these patients, 13,862 matched the inclusion criteria (age ≥ 15 years, blunt or penetrating trauma) for the derivation cohort. Nineteen patients were confirmed dead on ED arrival, and 10,580 were discharged from the ED or transferred to other medical facilities. A total of 3263 patients were finally included for analysis. A total of 12,403 out of 21,461 patients were included in the validation cohort. Fifty-three patients were confirmed dead on ED arrival, and 9244 were discharged from the ED or transferred to other medical facilities. A total of 3106 patients were finally included in the analysis (Fig. 2).

The patients in the derivation cohort had a median age of 60 (IQR, 46–73) years, and 66.0% were male. A total of 94.4% suffered from blunt trauma. The median SBP was 130 (IQR, 110–140) mmHg, the median RR was 20 (IQR, 18–20) per minute, the median GCS was 15 (IQR, 15–15) and the median SpO_2_ was 98% (IQR, 96% - 99%). The median RTS was 7.84 (IQR, 7.84–7.84), and the median ISS was 9 (IQR, 4–13). Overall, the in-hospital mortality rate was 11.3%. Table 2 shows the main demographic characteristics and physiological data of both groups.

**Table 2:** Characteristics of the derivation and validation groups

Characteristics	Derivation cohort	Missing case	Validation cohort	Missing case
	(*N* = 3263)	N, %	(*N* = 3106)	N, %
Age, yrs., median (IQR)	60 (46–73)	0 (0)	59 (45–72)^a^	0 (0)
Men (%)	2155 (66.0)	0 (0)	2065 (66.5)^b^	0 (0)
Trauma type, n (%)				
Blunt trauma	3079 (94.4)	0 (0)	2944 (94.8)	0 (0)
Penetrating trauma	184(5.6)	0 (0)	162 (5.2)	0 (0)
Physiological parameter, median (IQR)				
Systolic blood pressure (mmHg)	130 (110–140)	4 (0.1)	135 (118–150)^a^	0 (0)
Respiratory rate (per a minute)	20 (18–20)	4 (0.1)	20 (18–20)^a^	0 (0)
Glasgow Coma Scale	15 (15–15)	3 (0.1)	15 (15–15)	2 (0.1)
Oxygen saturation (%)	98 (96–99)	105 (3.2)	98 (96–99) ^a^	87 (2.8)
Trauma location, n (%)				
Head & neck injury	1057 (32.4)		1290 (41.5) ^b^	
Face injury	739 (22.6)		612 (19.7) ^b^	
Chest injury	857 (26.2)		804 (25.9)	
Abdomen injury	500 (15.3)		440 (14.2)	
Extremity injury	1383 (42.4)		1241 (40.0)	
RTS, median (IQR)	7.84 (7.84–7.84)	6 (0.2)	7.84 (7.84–7.84)	2 (0.1)
ISS, median (IQR)	9 (4–13)	289 (8.9)	9 (4–14)^a^	244 (7.9)
Death, n (%)	368 (11.3)	0 (0)	332 (10.7)	0 (0)

RTS Revised Trauma Score; ISS Injury Severity Score

^a^ *p* < 0.05 compared with derivation group using Mann-Whitney *U* test

^b^ *p* < 0.05 compared with derivation group using chi-square test

### Actual GCS vs. coded GCS value in the univariate analysis

The mortality prediction of the actual GCS score and the coded GCS value were also compared using univariate logistic regression. The OR of the actual GCS score was 1.62 (*p* < 0.001; 95% CI, 1.5647–1.6781). The ORs of the coded GCS scores of 3, 2, 1, and 0 were 8.56 (*p* < 0.001; 95% CI, 5.1683–14.1706), 22.77 (*p* < 0.001; 95% CI, 13.5975–38.1538), 60.87 (*p* < 0.001; 95% CI, 34.4921–107.4113), and 757.67(*p* < 0.001; 95% CI, 402.4326–1426.4840), respectively. These results were included to show which parameter was superior. After further analysis, we eventually chose to use the actual GCS score because it contributed to better calibration of the final model. While the conclusions were drawn in the final step of the analysis, we dropped the coded GCS value in next subsection.

### SpO_2_ vs. respiratory rate

The GCS score, SBP, RR and SpO_2_ were entered into the univariate logistic regression as continuous variables, and all of them showed significance. To evaluate the predictive ability of the multivariate model, RR and SpO_2_ was entered separately with GCS and SBP. The adjusted ORs of RR and SpO_2_ were 1.01 (*p =* 0. 974; 95% CI, 0.9648–1.0378) and 1.07 (*p* < 0.001; 95% CI, 1.0372–1.0956), respectively (Table 3).

**Table 3:** Univariate and multivariate analysis for Glasgow Coma Scale score, systolic blood pressure, respiratory rate, and oxygen saturation

	Univariate			Multivariate			Multivariate		
	OR	*P* value	95% CI	OR	*P* value	95% CI	OR	*P* value	95% CI
GCS	1.62	<0.001	1.5647–1.6781	1.57	<0.001	1.5132–1.6326	1.52	<0.001	1.4597–1.5766
SBP	1.03	<0.001	1.0289–1.0352	1.01	<0.001	1.0090–1.0183	1.01	0.008	1.0018–1.0120
RR	1.25	<0.001	1.2218–1.2850	1.00	0.974	0.9648–1.0378	-	-	-
SpO_2_	1.16	<0.001	1.1340–1.1858	-	-	-	1.07	<0.001	1.0372–1.0956

*OR* odds ratio; *CI* confidence interval; *GCS* Glasgow Coma Scale; *SBP* systolic blood pressure; *RR* respiratory rate; *SpO*_*2*_ peripheral oxygen saturation

### Multivariate analysis of SBP_NTS_, SpO_2NTS_, and actual GCS vs. SBP_NTS_, SpO_2NTS_, and coded GCS value

SBP_NTS_, SpO_2NTS_, and the actual GCS reached statistical significance in the multivariate logistic regression. The global predictive performance exhibited good discrimination (AUC = 0.935, *p* < 0.001; 95% CI, 0.9174–0.9526) and calibration (H-L χ ^2^ = 6.303, *p* = 0.178; Table 4). SBP_NTS_, SpO_2NTS_, and the coded value of GCS also showed significance but with less calibration than our final model (H-L χ ^2^ = 18.404, *p* = 0.001). The following equation was used to calculate Ps: Ps = 1 / (1 + e^-b^), b = −6.5406 + (0.4006 x GCS) + (0.2983 x SBP_NTS_) + (0.8709 x SpO_2NTS_). The final NTS model ranged from 1.2019 to 10.6867 and was calculated as follows:

**Table 4:** Results of logistic regression of GCS, SBP_NTS_, and SpO_2NTS_

	β	SE	*p*	95% CI	
Constant	- 6.5406	0.5805	0.000	−7.6786	−5.4027
GCS	0.4006	0.0196	0.000	0.3622	0.4391
SBP_NTS_	0.2983	0.0754	0.000	0.1504	0.4461
SpO_2NTS_	0.8709	0.1580	0.000	0.5611	1.1808

AUC = 0.935, Hosmer-Lemeshow χ^2^ = 6.303 (*p* = 0.178)

NTS = (0.4006 x GCS) + (0.2983 x SBP_NTS_) + (0.8709 x SpO_2NTS_).

### The NTS vs. the RTS, the MGAP, the GAP

The NTS showed better discrimination than the RTS (AUC = 0.935 vs. 0.917, respectively, AUC difference = 0.018, *p* = 0.001; 95% CI, 0. 0071–0. 0293) and a slightly lower AUC than the MGAP (AUC = 0.935 vs. 0.938, respectively, AUC difference = 0.003, *p* = 0.713; 95% CI, −0. 0114–0. 0166) and the GAP (AUC = 0.935 vs. 0.941, respectively, AUC difference = 0.006, *p* = 0.423; 95% CI, −0. 0080–0. 0190). In the validation cohort, the NTS showed better discrimination than the RTS, MGAP, and GAP (Table 5).

**Table 5:** Predictive performance of the NTS compared with the RTS, MGAP, and GAP in derivation and validation cohort

Score	Derivation cohort					Validation cohort				
	AUC	AUC difference	*p* value	95% CI		AUC	AUC difference	*p* value	95% CI	
NTS	0.935					0.919				
RTS	0.917	0.018	0.001	0.0071	0.0293	0.906	0.013	0.015	0.0009	0.0249
MGAP	0.938	0.003	0.713	−0.0114	0.0166	0.907	0.012	0.096	−0.0039	0.0271
GAP	0.941	0.006	0.423	−0.0080	0.0190	0.912	0.007	0.399	−0.0090	0.0224

### Observed mortality rate according to the NTS for triage (T-NTS) in complete data

**Fig. 3:**
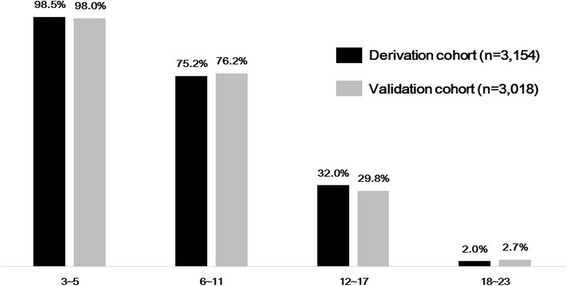
Observed mortality of low, intermediate, high, and very high risk groups categorized by New Trauma Score for triage in the derivation and validation cohorts

The T-NTS is calculated using the formula T-NTS = GCS + SBP_NTS_ + SpO_2NTS_. It ranges from 3 to 23. For triage, a T-NTS of 18, for which the sensitivity and specificity were 95% and 82%, respectively, was chosen to transfer patients to the trauma center. We categorized the patients into four groups: low (T-NTS 18–23), intermediate (T-NTS 12–17), high (T-NTS 6–11) and very high (T-NTS 3–5) risk for death. The observed mortality rates of the derivation cohort in each defined stratum were visualized along with those of the validation cohort in Fig. 3. The specificities of the RTS, the MGAP, and GAP were 80% (cutoff, RTS < 7.0), 80% (cutoff, MGAP score < 20), and 82% (cutoff, GAP score < 17), respectively.

## Discussion

The aim of our study was to develop a new trauma scoring system based on initial patient physiological data, the GCS score, SBP and SpO_2_. We found that the NTS significantly outperformed the RTS in mortality prediction of trauma patients but did not exceed the MGAP and GAP. At the fixed rate of 5% undertriage (sensitivity 95%), the NTS showed overtriage rate equal to or slightly lower than those of the RTS, MGAP and GAP in our study population. In the trauma setting, a high level of sensitivity rather than specificity for transferring patients to a specialized trauma center is essential to avoid patient deaths due to suboptimal care [8]. Increasing evidence shows that the overtriage of trauma patients leads to large wastes of socioeconomic and medical resources [26]. Lowering the overtriage rate with assurance of a convincing undertriage rate is important. According to our results, the specificity of the NTS at 95% sensitivity was over 82%, which is higher than RTS and MGAP and slightly lower than the GAP.

The MGAP and GAP may be superior to the NTS from some perspectives. For example, the components of the MGAP and GAP (mechanism, age, the GCS, and SBP) are immediately available at presentation, whereas the NTS needs SpO_2_. However, SpO_2_ measurement is not time-consuming or expensive in a modern trauma care system. In our study, the AUCs of the MGAP and GAP were larger than that of the NTS in the derivation cohort but were within the scope of statistical error. In the validation cohort, the AUCs of the MGAP and GAP were smaller than that of the NTS but were also within the range of statistical error. Despite the inconclusive results, the superiority of the NTS over the MGAP and GAP was to be expected; the MGAP and GAP already include age and mechanism, whereas the NTS comprises the same element used in the RTS. For this reason, only the NTS can be incorporated into the TRISS, which is the most widely used mortality prediction model for trauma patients. To date, no trauma scoring system has shown better performance than the TRISS. In addition to its use as a triage tool in clinical practice, the NTS may play an important role in trauma research due to its potential applicability as a substitute for the RTS in the TRISS.

We chose to use the actual GCS score rather than the coded GCS, which was initially proposed by Champion et al., in our prediction model. There is no firm evidence of an advantage of the GCS compared with the coded GCS. Among the severity scoring systems used for patients in the intensive care unit, the Acute Physiology and Chronic Health Evaluation (APACHE) uses the actual GCS [27], whereas the Simplified Acute Physiology Score (SAPS) and the Sequential Organ Failure Assessment (SOFA) use the coded GCS value [28, 29]. Most recently developed trauma scoring systems adopt the actual GCS, including the MGAP, GAP, Emergency Trauma Score (EMTRAS), BIG score (composed of base deficit, international normalized ratio, and the GCS), the UK Trauma Audit & Research Network prediction model, and the corticosteroid randomization after significant head injury (CRASH) model [5, 6, 30–32]. In our study, the actual GCS showed better calibration of the final model compared with the coded GCS.

The association between SBP and mortality in trauma patients has been assessed in many previous studies. Recent studies have recommended that the SBP threshold be increased up to 110 mmHg. Several authors have reported that elevated blood pressure was related to poor outcomes in traumatic brain injury (TBI). Additionally, Zafar et al. and Fuller et al. specifically mentioned that mortality in TBI showed a bimodal distribution [33–36]. In the general trauma population, the association between hypertension and mortality is not clearly known. However, during the initial period of trauma care, whether a given patient has TBI is not certain. To the best of our knowledge, there has been no study regarding this issue. In our population, hypertensive patients (SBP ≥ 150 mmHg) showed higher mortality than normotensive patients (SBP ≥ 110 mmHg; Fig 1c), and we believe this distribution might be caused by the relatively high proportion of head injury in the study patients (32.4% of the derivation cohort) (Table 2).

Only one study has assessed the possibility of replacing RR with SpO_2_ in the RTS (observational cohort study, prehospital setting, *n* = 1481) [37]. The authors concluded that RR and SpO_2_ do not add significant value to other variables in the RTS and TRISS. However, they said the power of the study might be insufficient to detect significance because of abundant missing data (approximately 35% of RR and SpO_2_). Although it was not advocated in the main conclusion of the study, SpO_2_ showed larger AUC for mortality compared with the RR (AUC, 0.747 vs. 0.691), and the SpO_2_ was more strongly correlated mortality than the RR was. Our study consistently showed that SpO_2_ is a better parameter than RR. A major concern regarding the use of SpO_2_ is non-measurability. We could not determine whether missing SpO_2_ values were caused by non-measurability or were simply missing in our patients. In terms of the retrospective analysis, we believe that this problem was not serious since we imputed the missing SpO_2_ on significant variables, including the ISS and outcome (in-hospital mortality). However, in clinical practice, non-measurable SpO_2_ leads to a failure to gain the final score. Most non-measurable SpO_2_ in trauma patients is associated with extremely low oxygenation or poor peripheral circulation caused by profound hemorrhagic shock, tension pneumothorax, cardiac tamponade, or cardiac arrest. Therefore, non-measurable SpO_2_ could be counted as zero (the lowest code value of the NTS) considering the patients’ symptoms, clinical appearance, and other physiological parameters.

Our study has some limitations. Of greatest concern is the selection bias. First, patients were transferred to our hospital from a relatively small area of approximately 60 km in radius. Our hospital is the only tertiary care center for major trauma in this area. Second, this was a single-center study, and therefore generalization could be erroneous. Accordingly, external validation in different populations and countries should be performed. Third, the target population was restricted to adults. Therefore, this score may not be useful for patients under 15 years of age. Because pediatric patients have unique physiological characteristics, separate studies are needed for the development and application of a trauma scoring system for the pediatric population.

## Conclusion

The NTS predicts in-hospital mortality substantially better than the RTS and not inferior to the MGAP and GAP. We hope that the NTS will be a useful tool for triage in trauma patients and will lead to an improvement in trauma management.

## Abbreviations

AIS: Abbreviated Injury Scales
AUC: areas under the receiver operating characteristic curve
CI: confidence interval
ED: emergency department
GAP: GCS, Age and Systolic Arterial Pressure
GCS: Glasgow Coma Scale
H-L: Hosmer-Lemeshow
ISS: Injury Severity Score
MGAP: Mechanism, GCS, and Age and Arterial Pressure
NTS: New Trauma Score
OR: odds ratio
Ps: predictive survival
ROC: receiver operating characteristic
RR: respiratory rate
RTS: Revised Trauma Score
SBP: systolic blood pressure
SBP_NTS_: code value of systolic blood pressure
SPo_2_: peripheral oxygen saturation
SPo_2NTS_: code value of peripheral oxygen saturation
T-NTS: New Trauma Score for Triage
TRISS: Trauma and Injury Severity Score
